# Effects of Physical Health, Mental Health, Social Environmental Factors, and Quality of Life on Social Participation of People With Physical Disabilities

**DOI:** 10.1002/brb3.70454

**Published:** 2025-05-05

**Authors:** Myoungsuk Kim, Seung Hee Ho, Hayeon Kim, Jaemin Park

**Affiliations:** ^1^ Program in Public Health Korea University Graduate School Seoul Republic of Korea; ^2^ National Rehabilitation Research Institute National Rehabilitation Center Seoul Republic of Korea

## Abstract

**Introduction:**

People with physical disabilities inevitably experience barriers to social participation, including physical limitations, health issues, stigma, discrimination, policies, and socio‐environmental factors. Although this is a critical public health issue, no multidimensional, comprehensive, evidence‐based intervention has previously addressed the specific factors affecting social participation in people with physical disabilities. This study explores the various factors influencing social participation, which contribute to health inequalities for people with physical disabilities. In particular, the physical and mental health, socio‐environmental factors, and quality of life (QoL) among people with physical disabilities are examined.

**Methods:**

The study used health data from surveys and clinical examinations conducted in 2021 and 2022 for 301 participants with physical disabilities in Korea. An observational, cross‐sectional analysis was performed using 47 independent variables to analyze the risk factors affecting social participation.

**Results:**

Crude analysis revealed 29 variables in demographic characteristics, disability characteristics, health behaviors, health status, mental health, socio‐environmental factors, and QoL that correlated significantly with social participation. The adjusted analysis revealed that difficulties in social participation were influenced in the following order: poor subjective health, lower QoL, inability to freely use public buildings in the community, lack of necessary income sources, difficulty in using transportation, selective eating habits, increased depression, and experiences of discrimination. The explanatory power of the regression model is 46.4%.

**Conclusion:**

To strengthen social participation, it is necessary to develop community programs that integrate rehabilitation treatment for managing physical disabilities and secondary health conditions with psychological and emotional support. In addition, social discrimination and health inequalities should be addressed through raising awareness and education about disabilities and participation.

## Graphical abstract

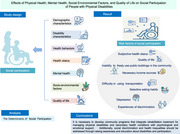

## 1 Introduction

Approximately 1.3 billion people, accounting for 16% of the global population, experience severe disabilities, and this number is steadily increasing (World Health Organization 2024). People with disabilities face higher rates of premature death, poorer health outcomes, and considerable limitations in daily life compared to others (World Health Organization 2024). They are more likely to encounter physical, socioeconomic, and policy‐related barriers than individuals without disabilities, which can affect various aspects of their lives (Hackett et al. 2020).

According to the WHO's recent “Global Report on Health Equity for Persons with Disabilities,” individuals with disabilities have the same right to the highest attainable standard of health as people without disabilities; however, they experience ableism, stigma, and discrimination in all aspects of life, and various forms of discrimination and exclusion in health systems, including unequal social conditions and environments, healthcare workers' lack of knowledge, bias among medical staff, discriminatory practices, inaccessible health facilities, and lack of information and data on disabilities (World Health Organization 2022).

Research has shown that disability status is associated with reduced participation in social activities (J. Kim et al. 2024). The social model of disability posits that an adverse social environment, characterized by factors such as discrimination and stigma (van Brakel et al. 2012) and inaccessible services and places (Smith et al. 2016), can reduce an individual's intrinsic desire to actively participate in society. Moreover, during the COVID‐19 pandemic, people with disabilities faced more severe health inequalities than people without disabilities, due to the increased risk of COVID‐19 infection, quarantine, adverse effects, and death (Courtenay and Perera 2020). According to the 2023 Panel Survey of Employment for Persons with Disabilities in South Korea, the percentage of respondents who stated they were “unable to participate in social activities” was 66.3% in 2020, 72.4% in 2021, 67.3% in 2022, and 69.6% in 2023 (Korea Employment Agency for Persons with Disabilities 2024).

The WHO's International Classification of Functioning, Disability and Health (ICF) integrates the traditional medical model and the social model, defining disability as the interaction between the individual's health status and environmental factors. Participation, a component of the ICF, refers to the extent to which individuals perform and participate in their roles in everyday life and social contexts. It defines limitations in activities and participation as forms of disability, and assesses how individuals are affected by environmental factors (World Health Organization 2001).

Therefore, this study investigated the factors affecting the social participation of people with physical disabilities using the participation domain of the World Health Organization Disability Assessment Schedule (WHODAS 2.0), a disability assessment tool developed based on WHO's ICF. In the WHODAS 2.0, participation is defined as “A person's involvement in a life situation. It represents the societal perspective of functioning” (Üstün et al. 2010). This refers to an individual's participation in life situations and represents a social perspective on functioning, encompassing how a person engages and interacts within society.

Among the types of disabilities, people with physical disabilities, particularly those with mobility impairments, are more likely to have limitations in community participation and social connectivity for various reasons, including physical environments, health problems, transportation barriers, and limited financial resources (Barclay et al. 2016; Hall et al. 2022). According to the Enforcement Decree of the Welfare of Persons with Disabilities Act of Korea (revised on December 31, 2018), a physical disability is defined as one that causes permanent damage to an individual's motor functions due to trauma to the limbs and trunk, deformity, paralysis, amputation due to lesions, and so on. It accounts for the largest proportion (43.7%) among the 15 types of disabilities in Korea as of 2023 (Korean Statistical Information Service 2024).

So far, studies on social participation have largely focused on specific areas, such as functional disability (Watanabe et al. 2024), frailty (Sone et al. 2023), quality of life (QoL) (Jespersen et al. 2019), socioeconomic factors (Melo and Valdes 2011), health conditions (Hreha et al. 2019), and mental health (Choi et al. 2021). Comprehensive, multidimensional research on the various factors influencing social participation has not been conducted.

This study explored various factors affecting social participation that contribute to health inequalities among people with disabilities using health cohort data for individuals with physical disabilities in South Korea. It adopts a comprehensive approach based on the ICF framework, considering components such as physical function, activity, participation, and environmental and personal factors. The analysis includes demographic and disability characteristics, health behaviors, physical and mental health, social and environmental factors, and QoL aspects. The findings aim to offer policy implications and foundational data for developing disability support programs, promoting the social inclusion of individuals with physical disabilities.

## 1 Materials and Methods

### 1.1 Study Population

Data were extracted from the cross‐sectional data of respondents who participated in the 2021/2022 survey of the Korean Health Cohort Study for People with Physical Disabilities conducted by the National Rehabilitation Center under the Ministry of Health and Welfare. This study included data from a total of 301 cohort registrants after excluding dropouts. The participants were residents of Seoul who had been diagnosed with a physical disability, were aged 40–69 years, had scored 24 or above on the Korean version of the Mini‐Mental State Examination (K‐MMSE) (proxy respondents for those scoring below 24).

The research process involved collecting data by conducting a questionnaire on the subjects, then conducting clinical examinations to measure body measurements, blood pressure, pulse, body composition analysis, blood and urine tests, electrocardiograms, chest examinations, and bone density tests. The Korean Health Cohort Study for People with Physical Disabilities was approved by the Institutional Review Board of the National Rehabilitation Center (NRC‐2015‐04‐034). Written consent was obtained from all participants before the study began.

### 1.2 Measures

#### 1.2.1 Social Participation

Social participation, the dependent variable in this study, was measured using the participation domain of the WHODAS 2.0, which includes the following evaluation areas: cognition, mobility, self‐care, getting along, life activities, and participation. The participation domain comprises eight items and the scores assigned to each item—none (1), mild (2), moderate (3), severe (4), extreme (5)—were summed. Higher scores indicated greater difficulty in participating in social activities (Üstün et al. 2010).

#### 1.2.2 Sociodemographic and Disability Characteristics

The participants' sociodemographic characteristics were gender, age, marital status, education level, occupation, and income. Income is the average monthly income of a household, including all incomes combined. The disability characteristics were defined by the primary disability and its severity. The primary disability was classified based on the participant's main diagnosis, which included categories such as polio, spinal cord injury (SCI) and disease, and other physical disabilities.

#### 1.2.3 Health Behavior and Health Status

Regular exercise and moderate physical activity were categorized into “yes” or “no.” The categories for “regular daily meals” and “unrestricted food preferences” were divided into “yes,” “average (sometimes),” and “no.” For moderate physical activity, the question “In the past week, how many days did you engage in moderate physical activity for at least 10 min, where you felt slightly tired or out of breath?” was changed into a “yes or no” question.

Weight control efforts were measured by asking if participants had tried to manage their weight over the past year, with responses categorized as “yes” or “no.” Subjective oral health was rated as “good,” “average,” or “poor,” while chewing discomfort was classified as “no discomfort,” “moderate,” or “discomfort.” “Yes” and “no” variables were used for fall experience, fear of falling, chronic pain, hypertension, diabetes, obesity, and osteoporosis. A fall experience referred to an incident within the past year, including slipping, tripping, collapsing, or falling. Chronic pain was defined as pain lasting for more than 30 days within the past 3 months. The presence of chronic conditions (hypertension, diabetes, obesity, and osteoporosis) was determined through clinical tests (blood pressure, blood glucose, BMI, and bone density) or prior diagnosis and medication use.

Activities of Daily Living (ADL) and Instrumental Activities of Daily Living (IADL) were categorized as either “able to perform” or “unable to perform.” Each item was measured on a three‐point scale: “able to do independently without assistance,” “able to do with partial assistance,” and “unable to do without assistance.” Being able to perform a task independently was considered “able to perform,” whereas requiring any level of assistance was classified as “unable to perform independently.”

#### 1.2.4 Mental Health

Stress was categorized as “not felt,” “average,” and “felt,” whereas suicidal thoughts were classified as either “present” or “absent.” Depression was measured using the Korean version of the 20‐item Center for Epidemiologic Studies Depression Scale (Radloff 1977), which asks about the respondent's feelings over the past week. Each item was rated on a four‐point scale (0 = rarely; 3 = most of the time), with a maximum score of 60 points. Higher scores indicated a greater tendency toward depression.

Current life satisfaction was categorized as “satisfied,” “average,” or “dissatisfied,” whereas subjective health status was classified as “good,” “average,” or “poor.” Cognitive function was assessed using the K‐MMSE, with a maximum score of 30 points. Scores of 24 and above indicated normal cognitive function, scores of 18–23 indicated mild cognitive impairment, and scores of 17 or below indicated severe cognitive impairment (Kang et al. 1997; J. M. Kim et al. 2003).

#### 1.2.5 Social and Environmental Factors

The items belonging to the social environmental factors are divided into 14 items in total, categorized as “yes” or “no,” and include the following items: “I have difficulty using the transportation that I want, I have difficulty demonstrating my professional abilities in my environment, I am treated without discrimination compared to nondisabled people with similar abilities, I have secured a source of income necessary for my living, I have people I meet constantly, there are groups or organizations that can give me a sense of belonging, my family fully accepts my disability, and I am satisfied with my family.”

#### 1.2.6 Quality of Life

QoL was measured using the EuroQol‐5 Dimension (EQ‐5D), which is one of the most widely used generic index measures of health‐related quality of life (HrQoL) (Lee et al. 2009). The EQ‐5D is a standardized health status measurement tool developed by the EuroQol group and is widely used to assess HrQoL (Rabin and de Charro 2001).

The EQ‐5D rates five dimensions of mobility, self‐care, usual activities, pain/discomfort, and anxiety/depression into three levels of health (no problem, some problems, and severe problems). This study assigned weighted values to each of the five dimensions, with scores ranging from 1 (indicating perfect health) to −0.171 (the lowest possible score). Therefore, a comprehensive measure of HrQoL was obtained (Lee et al. 2009).

$$\begin{array}{ccc} \mathrm{EQ}-5D & = & 1-(0.050+0.096\;\times \mathrm{MO}2+0.418\;\times \;\mathrm{MO}3+0.046\\ & & \;\times \;\mathrm{SC}2+0.136\;\times \;\mathrm{SC}3+0.051\;\times \;\mathrm{UA}2+0.208\;\times \;\mathrm{UA}3\\ & & +\;0.037\;\times \mathrm{PD}2+0.151\;\times \;\mathrm{PD}3+0.043\;\times \;\mathrm{AD}2+0.158\\ & & \;\times \;\mathrm{AD}3+0.050\;\times \;N3). \end{array}$$

Where MO2: mobility, level 2; MO3: mobility, level 3; SC2: self‐care, level 2; SC3: self‐care, level 3; UA2: usual activities, level 2; UA3: usual activities, level 3; PD2: pain × discomfort, level 2; PD3: pain × discomfort, level 3; AD2: anxiety × depression, level 2; AD3: anxiety × depression, level 3; N3: indicates whether any dimension is at level 3.

### 1.3 Statistical Analysis

In the crude analysis, descriptive statistics were performed to identify the general characteristics of the participants. To examine the associations and correlation between each independent variable and social participation, Student's *t*‐test, Kruskal–Wallis test, and Pearson's correlation analysis were utilized (Tables 1, 2, 3, 4). Next, to identify the factors influencing social participation among people with physical disabilities, multiple linear regression analysis was conducted as a multivariable analysis (Table 5).

**TABLE 1:** Relationship between social participation and demographic/disability characteristics (*N* = 301).

Characteristics	Classification	*N* (%)	Mean	±SD	*t, H*
Gender	Male	140 (46.5)	16.97	±6.65	−2.295*
	Female	161 (53.5)	18.61	±5.73	
Age^a^	40–49	15 (5.0)	15.60	±5.45	2.338
	50–59	104 (34.5)	18.25	±6.28	
	60–69	145 (48.2)	17.92	±6.06	
	70 and over	37 (12.3)	17.32	±6.96	
Marital status^a^	Married	168 (55.8)	17.85	±6.41	1.402
	Single	59 (19.6)	18.63	±6.49	
	Other (divorced, widowed, or cohabiting)	74 (24.6)	17.23	±5.52	
Education level^a^	Bachelor's or higher	87 (28.9)	17.62	±6.14	0.836
	High school graduate	101 (33.6)	17.49	±5.92	
	Middle school graduate	51 (16.9)	18.22	±6.12	
	Elementary school graduate or lower	62 (20.6)	18.45	±6.93	
Employment	Yes	146 (48.5)	16.60	±5.80	−3.430**
	No	155 (51.5)	19.02	±6.39	
Income^a^	More than 3.01 million KRW	48 (17.6)	15.92	±5.75	11.379*
	2.01–3 million KRW	30 (11.0)	16.40	±5.62	
	1.01–2 million KRW	85 (31.3)	16.88	±5.77	
	Less than 1 million KRW	109 (40.1)	18.98	±6.37	
Primary disability^a^	Poliomyelitis	134 (44.5)	18.24	±6.62	1.812
	Spinal cord injury or disease	56 (18.6)	18.36	±6.42	
	Other physical disabilities	111 (36.9)	17.12	±5.56	
Severity of disability	Mild	86 (28.6)	16.51	±5.85	−2.375*
	Severe	215 (71.4)	18.38	±6.30	

*Note*: Student's *t*‐test was used for analysis; missing values (*n*): income (29).

^a^Kruskal–Wallis test (*H*).

*p < 0.05, **p < 0.01.

**TABLE 2:** Relationship between social participation and health behaviors/status (*N* = 301).

Characteristics	Classification	*N* (%)	Mean	±SD	*t, H*
Regular exercise	Practiced	173 (57.5)	17.74	±5.96	−0.347
	Not practiced	128 (42.5)	17.99	±6.57	
Moderate physical activity	Practiced	103 (34.2)	18.48	±5.98	1.266
	Not practiced	198 (65.8)	17.52	±6.33	
Regular daily meals^a^	Yes	148 (49.2)	17.70	±6.17	0.287
	Sometimes (occasionally)	78 (25.9)	18.21	±5.91	
	No	75 (24.9)	17.77	±6.68	
No selective eating^a^	Yes	186 (61.8)	17.15	±6.20	9.121*
	Sometimes (occasionally)	74 (24.6)	18.18	±5.49	
	No	41 (13.6)	20.41	±6.95	
Sleep duration^a^	7 h or more	127 (42.2)	16.83	±6.37	8.145*
	Between 6 and 7 h	87 (28.9)	18.10	±5.65	
	Between 5 and 6 h	57 (18.9)	18.72	±6.43	
	Less than 5 h	30 (10.0)	19.73	±6.28	
Subjective body shape perception^a^	On the slim side	42 (14.0)	17.71	±6.71	1.867
	Average	109 (36.2)	17.30	±6.23	
	Obese	150 (49.8)	18.28	±6.08	
Efforts for weight control	Makes effort	227 (75.4)	18.24	±5.94	1.777
	No effort	74 (24.6)	16.65	±6.91	
Subjective oral health^a^	Good	53 (17.6)	18.02	±6.08	0.588
	Average	135 (44.9)	17.42	±5.89	
	Bad	113 (37.5)	18.27	±6.67	
Chewing discomfort^a^	Not uncomfortable	152 (50.5)	17.59	±6.24	3.652
	Average	50 (16.6)	16.60	±5.46	
	Uncomfortable	99 (32.9)	18.87	±6.45	
Experience falling	None	133 (44.2)	17.20	±6.16	−1.603
	Yes	168 (55.8)	18.36	±6.24	
Fear of falling	None	78 (25.9)	15.13	±6.38	−4.637***
	Yes	223 (74.1)	18.80	±5.88	
Chronic pain	None	154 (51.2)	16.08	±5.79	−5.273***
	Yes	147 (48.8)	19.70	±6.13	
High blood pressure	None	129 (42.9)	17.55	±6.17	−0.717
	Yes	172 (57.1)	18.07	±6.27	
Diabetes	None	220 (73.1)	17.73	±6.12	−0.551
	Yes	81 (26.9)	18.17	±6.52	
Obesity	None	155 (51.8)	17.52	±6.18	−0.828
	Yes	144 (48.2)	18.11	±6.24	
Osteoporosis	None	213 (71.5)	17.40	±6.13	−2.026*
	Yes	85 (28.5)	19.01	±6.33	
ADL	Able to perform independently	177 (58.8)	16.18	±5.78	−5.877***
	Unable to perform independently	124 (41.2)	20.23	±6.06	
IADL	Able to perform independently	129 (42.9)	15.67	±5.33	−5.637***
	Unable to perform independently	172 (57.1)	19.48	±6.35	

*Note*: Student's *t*‐test was used for analysis; missing values (*n*): obesity (2), osteoporosis (3).

^a^Kruskal–Wallis test (*H*).

*p < 0.05, ***p < 0.001.

**TABLE 3:** Relationship between social participation and mental health (*N* = 301).

Characteristics	Classification	*N* (%)	Mean	±SD	*t, H, r*
Perceived stress^a^	Not felt	71 (23.6)	15.11	±5.63	22.034***
	Average	118 (39.2)	18.05	±6.07	
	Felt	112 (37.2)	19.37	±6.21	
Depression assessment (CES‐D)	—	301 (100.0)	10.29	±11.05	0.377***
Suicidal thoughts***	None	242 (80.4)	17.19	±6.07	−3.770***
	Yes	59 (19.6)	20.53	±6.15	
Current life satisfaction^a^	Satisfied	162 (53.8)	15.73	±5.55	51.375***
	Average	102 (33.9)	19.02	±5.35	
	Dissatisfied	37 (12.3)	23.86	±6.55	
Subjective health status^a^	Good	84 (27.9)	14.73	±5.39	64.627***
	Average	81 (26.9)	15.99	±5.11	
	Bad	136 (45.2)	20.88	±5.93	
K‐MMSE^a^	Normal	290 (96.3)	17.84	±6.10	0.992
	Mild cognitive impairment	8 (2.7)	16.25	±7.50	
	Severe cognitive impairment	3 (1.0)	22.33	±13.58	

*Note*: Student's *t*‐test was used for analysis.

Abbreviation: K‐MMSE: Korean version of the Mini‐Mental State Examination.

^a^Kruskal–Wallis test (*H*), Pearson's correlation analysis.

***p < 0.001.

**TABLE 4:** Relationship between social participation and social and environmental factors/quality of life (*N* = 301).

Characteristics	Classification	*N* (%)	Mean	±SD	*t, r*
Free access to community public buildings	Yes	208 (69.1)	16.43	±5.39	−5.770***
	No	93 (30.9)	21.02	±6.78	
Securing means of usage when needed	Yes	246 (81.7)	17.24	±5.90	−3.329**
	No	55 (18.3)	20.58	±6.91	
Difficulty using transportation	No	166 (55.1)	16.17	±5.69	−5.434***
	Yes	135 (44.9)	19.91	±6.24	
Difficulty in displaying professional abilities due to the environment	No	146 (48.5)	16.73	±6.03	−3.059**
	Yes	155 (51.5)	18.90	±6.23	
Equal treatment without discrimination	Yes	222 (74.0)	16.73	±5.69	−4.960***
	No	78 (26.0)	20.90	±6.60	
Adequate medical services	Yes	235 (78.1)	17.02	±5.82	−4.509***
	No	66 (21.9)	20.80	±6.72	
Securing necessary sources of income	Yes	169 (56.1)	16.05	±5.68	−5.982***
	No	132 (43.9)	20.14	±6.14	
Closeness with fellow persons with disabilities	Yes	258 (85.7)	17.62	±6.02	−1.528
	No	43 (14.3)	19.19	±7.25	
Having many nondisabled friends	Yes	220 (73.1)	16.84	±5.81	−4.793***
	No	81 (26.9)	20.58	±6.50	
Ongoing interpersonal relationships	Yes	268 (89.0)	17.60	±6.03	−1.938
	No	33 (11.0)	19.82	±7.38	
Affiliation with organizations or institutions	Yes	236 (78.4)	17.38	±5.95	−2.498*
	No	65 (21.6)	19.54	±6.91	
Support from family	Yes	259 (87.2)	17.61	±6.08	−2.304*
	No	38 (12.8)	20.08	±6.79	
Family's acceptance of the disability	Yes	266 (89.6)	17.45	±5.97	−3.919***
	No	31 (10.4)	21.97	±6.87	
Satisfaction with family	Satisfied	257 (86.5)	17.59	±6.23	−2.398*
	Dissatisfied	40 (13.5)	20.10	±5.73	
EQ‐5D ***		301 (100.0)	0.70	±0.20	−0.474***

*Note*: Student's *t*‐test was used for analysis; EQ‐5D = 1 − (0.050 + 0.096 × MO2 + 0.418 × MO3 + 0.046 × SC2 + 0.136 × SC3 + 0.051 × UA2 + 0.208 × UA3 + 0.037 × PD2 + 0.151 × PD3 + 0.043 × AD2 + 0.158 × AD3 + 0.050 × N3) Where MO2: mobility, level 2; MO3: mobility, level 3; SC2: self‐care, level 2; SC3: self‐care, level 3; UA2: usual activities, level 2; UA3: usual activities, level 3; PD2: pain × discomfort, level 2; PD3: pain × discomfort, level 3; AD2: anxiety × depression, level 2; AD3: anxiety × depression, level 3; N3: indicates whether any dimension is at level 3. Missing values (*n*): family support, family disability acceptance, satisfaction with family (4); no family.

*p < 0.05, **p < 0.01, ***p < 0.001.

**TABLE 5:** Risk factors that influence social participation (*N* = 301).

	Unstandardized coefficients		Standardized coefficients	*t*	Tolerance	VIF
	*B*	SE	*Β*			
(Constant)	16.712	1.541		10.847		
CES‐D	0.076	0.029	0.137	2.656**	0.793	1.261
Subjective health status (bad)	3.015	0.624	0.247	4.827***	0.807	1.239
No selective eating (no)	2.475	0.826	0.141	2.998**	0.959	1.042
Free access to community public buildings (no)	2.406	0.646	0.183	3.723***	0.875	1.143
Difficulty using transportation (yes)	1.814	0.591	0.148	3.068**	0.906	1.104
Equal treatment without discrimination (no)	1.725	0.667	0.126	2.585*	0.897	1.115
Securing necessary sources of income (no)	1.869	0.599	0.153	3.118**	0.886	1.128
EQ5D	−6.103	1.721	−0.191	−3.546***	0.732	1.367

*Note: F*(*p*) = 27.340(0.000), *R* ^2^ = 0.464, adj.*R* ^2^ = 0.447, Durbin–Watson = 2.031. EQ‐5D = 1 − (0.050 + 0.096 × MO2 + 0.418 × MO3 + 0.046 × SC2 + 0.136 × SC3 + 0.051 × UA2 + 0.208 × UA3 + 0.037 ×PD2 + 0.151 × PD3 + 0.043 × AD2 + 0.158 × AD3 + 0.050 × N3). Where MO2: mobility, level 2; MO3: mobility, level 3; SC2: self‐care, level 2; SC3: self‐care, level 3; UA2: usual activities, level 2; UA3: usual activities, level 3; PD2: pain × discomfort, level 2; PD3: pain × discomfort, level 3; AD2: anxiety × depression, level 2; AD3: anxiety × depression, level 3; N3: indicates whether any dimension is at level 3.

*p < 0.05, **p < 0.01, ***p < 0.001.

Statistical significance for all analyses was set at *α* < 0.05. Missing data were excluded from analyses (except for income of 9.6%, other missing values ≤ 1.3%). SPSS V. 27(IBM Corp.) was used to conduct all analyses.

## 2 Results

The average social participation score of the participants was 17.85 out of a maximum of 40 points. Univariate analysis showed significant differences in the relationship between social participation and sociodemographic and disability characteristics depending on gender, occupation, income, and disability severity. Women, those without a job, those with lower incomes, and individuals with more severe disabilities experienced greater difficulties in social participation (Table 1).

In the health behavior and health status domains, factors such as having a balanced diet, sleep duration, fear of falling, chronic pain, osteoporosis, ADL, and IADL were significant.

Those who do not have a balanced diet, sleep less, have a fear of falling, have chronic pain and osteoporosis, or are unable to perform ADL and IADL independently face more difficulties in social participation (Table 2). In the mental health domain, perceived stress, depression (CES‐D), suicidal thoughts, current life satisfaction, and subjective health status were significantly related to the social participation score. Higher difficulty in social participation was observed among individuals experiencing stress, higher depression levels, suicidal thoughts, dissatisfaction with life, and poor subjective health status (Table 3).

Regarding social and environmental factors, difficulties in social participation increased when individuals could not freely use public buildings in their community, unable to secure necessary means of utilization, had trouble using transportation, found it hard to demonstrate their professional abilities due to environmental constraints, experienced discrimination, did not receive appropriate medical services, lacked necessary income sources, didn't have many nondisabled friends, did not belong to any groups or organizations providing a sense of belonging, lacked family support or acceptance of their disability, were dissatisfied with their family, or had lower EQ‐5D scores (Table 4).

The results of the adjusted analysis using multiple linear regression showed that the regression model was statistically significant (*F* = 27.340, *p* < 0.000), and the explanatory power of the regression model was 46.4%. The Durbin–Watson statistic was 2.031, suggesting no issues with the independence of errors, and the variance inflation factor indicated no multicollinearity problems. The variables of the final model, selected through stepwise selection, included CES‐D, subjective health status, unrestricted diet, free use of public community buildings, difficulty using transportation, nondiscriminatory treatment, securing necessary income, and EQ‐5D. All these variables were statistically significant. Social participation difficulties increased with higher depression scores (CES‐D), poorer subjective health, selective eating habits, difficulty accessing public community buildings, trouble using transportation, experiencing discrimination compared to nondisabled individuals with similar abilities, and not securing the necessary income. By contrast, higher EQ‐5D scores (indicating better QoL) were associated with lower social participation difficulties, suggesting a positive impact on social participation.

Comparing the magnitude of influence on factors related to social participation, the order was subjective health status, QoL, free access to community public buildings, securing necessary sources of income, difficulty using transportation, no selective eating, depression, and equal treatment without discrimination (Table 5).

## 3 Discussion

Previous studies have explored the relationship between disability and social participation or the impact of specific factors on social participation and social relationships. However, the complex interplay of various elements affecting the low levels of social participation among individuals with disabilities is unclear. Therefore, this study used 47 independent variables across dimensions of physical health, mental health, social and environmental factors, and QoL to assess their impact on social participation of people with disabilities, using data from a health cohort of people with physical disabilities in South Korea.

Pearson's correlation analysis between social participation and CES‐D showed a highly significant positive correlation (*r* = 0.377, *p* < 0.001), indicating that depression increased with an increase in social participation difficulty. Meanwhile, Pearson's correlation analysis between social participation and EQ‐5D showed a highly significant negative correlation (*r* = −0.474, *p* < 0.001), indicating that increase in difficulties in social participation, decreased QoL (EQ‐5D).

The analysis of factors influencing social participation for all independent variables showed that the regression model's explanatory power was 46.4%. Individuals experienced more difficulties in social participation when they were more depressed, had poorer subjective health, had selective eating habits, faced difficulties in freely accessing public buildings in the community, had trouble using desired transportation, experienced discrimination compared to nondisabled individuals with similar abilities, or failed to secure income sources necessary for living. By contrast, the higher QoL index (EQ‐5D) more positively influenced social participation.

The findings of this study align with previous research that has presented risk factors for social participation among people with disabilities in a fragmented manner. It is also consistent with the research results that there are multiple factors that hinder social participation of people with physical disabilities who have mobility impairments (Hall et al. 2022). Barclay et al. (2015) confirmed that the barriers to participation for people with mobility impairments include health problems and a lack of financial resources, and that people with physical disabilities are at a higher risk of experiencing inaccessible physical environments than people with other types of disabilities. These people may be unable to participate frequently in local community activities held in inaccessible areas (Macdonald et al. 2018). The ability to participate in meaningful life roles and activities outside the home can change and diminish, particularly after SCIs (Brown et al. 2002; Whiteneck et al. 2004). Adequate financial resources and social support (from friends, family, and peer mentors) helped people with SCI participate in social activities, whereas physical environments, unsupportive social attitudes, and mental health problems were identified as barriers to community participation (Barclay et al. 2015).

Due to severe psychological challenges after an accident, physical disabilities, which involve the experience of declining physical function, make it difficult for individuals to engage in employment, economic activities, leisure activities, and self‐care (H. Kim 2023). Unemployment and poor health among people with mobility impairments are both related to a decrease in social participation (Hall et al. 2022), whereas employment was related to health improvements for people with disabilities (Scelza et al. 2005).

People with acquired physical disabilities experience more severe stress in accepting and overcoming their disabilities and face greater psychological and emotional challenges compared to those with congenital disabilities (Priestley 2003). Furthermore, depression is significantly related to people with SCI participating in social roles, which may be due to the complex relationship between depression and social skills or the limitations in engaging in basic social roles (Fekete et al. 2017). Johnston et al. (2007) argue that intervention approaches considering negative emotional aspects enhance perception levels during the recovery process and positively influence their limitations on activities and participation. Accordingly, emotional level is one of the key factors in improving levels of social participation and requires lots of attention as it can serve as a foundation for social disabilities (Scott et al. 2012).

In addition, people with long‐term physical disabilities who experience secondary health conditions (SHCs) have significantly reduced enjoyment of daily activities and social participation, limiting their engagement in social activities, which can decrease their QoL (Hreha et al. 2019). Social participation has been shown to have a significant impact on the QoL of people with disabilities in previous studies, as shown in the results of this study (*p* < 0.001). Acceptance, understanding, discrimination and prejudice from society, and the ability to participate in society are important aspects of an individual's QoL across all disability categories. Baumeister and Leary (1995) state that “humans are fundamentally and universally motivated by a need for belonging, that is, a strong desire to form and maintain enduring interpersonal attachments.”

Jespersen et al. (2019) argue that the QoL among adults with disabilities is influenced by environmental factors, including social participation, social relationships and connections, and life satisfaction, which is also consistent with the findings of this study. Therefore, it is important to strengthen social relationships and improve the environment to enhance QoL and social participation.

Meanwhile, a recent study of a group of individuals with physical disabilities found that SHC (e.g., chronic musculoskeletal pain, spasticity, fatigue, depression, or sleep disturbance) reduced self‐reported satisfaction regarding social role participation (Battalio et al. 2018). Similarly, in the crude analysis of this study, osteoporosis and fear of falling emerged as significant variables. People with a disability due to an early‐onset health condition need to manage the physical impairment and SHCs associated with the primary disability to participate in society.

The significance of this study lies in its focus on the social health of individuals with physical disabilities while identifying key factors—such as health behaviors, physical and mental health status, economic, environmental, social factors, and QoL—that influence their social participation. This study suggests evidence‐based interventions to promote the social participation of people with physical disabilities. However, despite the importance of these findings, the relatively small sample size makes it difficult to generalize the findings to the broader population of individuals with physical disabilities in South Korea. In addition, this study did not apply corrections for multiple comparisons due to the nature of exploratory analysis, and therefore the possibility of Type I error cannot be completely ruled out. Future studies need to consider these limitations to further strengthen the reliability of the findings.

## 4 Conclusion

Based on the health cohort data of individuals with physical disabilities in South Korea, an in‐depth analysis of the risk factors affecting the social participation of persons with physical disabilities during the COVID‐19 pandemic identified that subjective health status, QoL, free access to community public buildings, securing necessary resources, transportation use, no selective eating, depression, and nondiscriminatory treatment were significant factors influencing their social participation. This finding has crucial policy implications. Social participation is an important indicator of an individual's social well‐being and is closely linked to QoL. To enhance social participation, community programs that integrate rehabilitation for managing physical disabilities and SHCs with psychological and emotional support must be developed. In addition, providing education to improve awareness about disabilities and participation is essential to reduce social discrimination and health inequalities.

## Author Contributions

**Myoungsuk Kim**: conceptualization, methodology, software, data curation, investigation, validation, formal analysis, supervision, visualization, project administration, writing – original draft, writing – review and editing. **Seung Hee Ho**: supervision, resources, funding acquisition, validation, project administration. **Hayeon Kim**: supervision, investigation. **Jaemin Park**: investigation.

### Peer Review

The peer review history for this article is available at https://publons.com/publon/10.1002/brb3.70454.

## Data Availability

Research data are not shared.
